# Identifying aging-related genes in mouse hippocampus using gateway nodes

**DOI:** 10.1186/1752-0509-8-62

**Published:** 2014-05-27

**Authors:** Kathryn M Dempsey, Hesham H Ali

**Affiliations:** 1 Department of Pathology & Microbiology, University of Nebraska Medical Center, Omaha, USA; 2 College of Information Science and Technology, University of Nebraska at Omaha, Omaha, USA

**Keywords:** Correlation networks, Klotho, Gateway node, Aging-related genes, Hippocampus

## Abstract

**Background:**

High-throughput studies continue to produce volumes of metadata representing valuable sources of information to better guide biological research. With a stronger focus on data generation, analysis models that can readily identify actual signals have not received the same level of attention. This is due in part to high levels of noise and data heterogeneity, along with a lack of sophisticated algorithms for mining useful information. Networks have emerged as a powerful tool for modeling high-throughput data because they are capable of representing not only individual biological elements but also different types of relationships en masse. Moreover, well-established graph theoretic methodology can be applied to network models to increase efficiency and speed of analysis. In this project, we propose a network model that examines temporal data from mouse hippocampus at the transcriptional level via correlation of gene expression. Using this model, we formally define the concept of “gateway” nodes, loosely defined as nodes representing genes co-expressed in multiple states. We show that the proposed network model allows us to identify target genes implicated in hippocampal aging-related processes.

**Results:**

By mining gateway genes related to hippocampal aging from networks made from gene expression in young and middle-aged mice, we provide a proof-of-concept of existence and importance of gateway nodes. Additionally, these results highlight how network analysis can act as a supplement to traditional statistical analysis of differentially expressed genes. Finally, we use the gateway nodes identified by our method as well as functional databases and literature to propose new targets for study of aging in the mouse hippocampus.

**Conclu**s**ions:**

This research highlights the need for methods of temporal comparison using network models and provides a systems biology approach to extract information from correlation networks of gene expression. Our results identify a number of genes previously implicated in the aging mouse hippocampus related to synaptic plasticity and apoptosis. Additionally, this model identifies a novel set of aging genes previously uncharacterized in the hippocampus. This research can be viewed as a first-step for identifying the processes behind comparative experiments in aging that is applicable to any type of temporal multi-state network.

## Background

High-throughput assays have become a staple of biological research; however the volume of data available is difficult to analyze without automation. Experiments that examine an entire cellular system at single or multiple states (where a state can refer to a time point, disease stage, or healthy versus diseased environment) require not only computational power, but also efficient algorithms and data models that are able to deliver reliable analyses in a short amount of time. Further, the recent inception of data-driven [1] bioinformatics has evinced a new landscape of research requiring analytic methods that can handle massive, heterogeneous datasets. For example, as the costs of sequencing sinks and the demand for personalized genomics rises, the data will become even more multidimensional; the concept of “long data,” or data that is sampled repeatedly over a long period of time, is already collected for analysis in social media, advertising, and sales markets [2]; it is only a matter of time before this concept is embraced by high throughput bioinformatics studies. Thus, there is and will continue to be a growing need for technologies and benchmarks in this new landscape of data-driven bioinformatics.

Network representation is becoming a popular tool for modeling these types of data-driven experiments; for gene expression analysis, network models are able to store and showcase relationships between genes and gene products. Statistical functional enrichments can then be performed based on structural aspects of these relationships, whereas in traditional statistical analyses, capturing enrichments based on gene relationships is difficult at best [3]. Further, application of graph theoretic concepts to biologically data-driven networks has been shown to readily identify structures that can be directly tied to the mechanisms behind cellular function in biological networks such as the protein-protein interaction network [4] and the metabolome [5], among others [6]. The years of study spent on graph theory have optimized the efficiency of commonly used algorithmic network algorithms, making the marriage between graph theory and the large network model a natural next step in network analysis. Our research here probes the effectiveness of graph theoretic modeling/application by identifying key structural nodes in a dual-state temporal correlation network made from high-throughput data. It has been observed that in a modular correlation network, certain genes connect clusters between different states (i.e. stage I vs. stage II) [7] but the clusters connected by those genes rarely share more than a handful of genes. Those genes, serving almost as a pivot between two states thus become interesting targets of study in this research as they tend to link large, dense clusters of genes, and would serve as a readily available and reproducible candidate in many publicly available gene expression datasets. The research described here proposes a formal definition of these pivot or “gateway” genes based on the degree of a node in a 2-state network. A proof-of-concept is presented using expression data from the hippocampus of C57/Bl6 mice at 2 months and 16 months; the results of which suggest that these gateway genes provide insight into what drives the aging process in the murine brain.

### Network structure & analysis

Network structure has been tied to cellular function from since the discovery of the link between degree and essential proteins in the yeast interactome [8]. Initial studies performed on protein-protein interaction networks indicated that these networks adhered to the power-law degree distribution, meaning that many nodes in the network are poorly connected and a few nodes are very well connected; these nodes are known informally as “hubs [4,8]”. Hubs have been found in the yeast protein-protein interaction network (also known as an interactome) to correspond to essential genes [8] and have been found to be critical for maintenance of structure in other biological networks as well, such as the metabolome [5] and the correlation network [9-11]. Clustering coefficient can point toward the modularity of the network [4], and previous studies to identify modules in clustered networks indicate that when found, tend to correspond to genes or gene products working together toward some discrete function, such as a protein complex in an interactome [4] or as a regulatory cohort [12]. Many algorithms currently exist that are able to find clusters within networks that employ clustering via random seed selection and growing, spectral clustering, or clustering coefficient [13-15]. It is worth nothing that while gene clusters tend to correspond to biological functions, the actual structures they form in the network can be mined based solely on network structure, often without the help of biological annotation data**.** Thus, the link between network structure and function can be exploited to identify known and unknown network elements.

While other forms of biological networks have been found to be modular and scale-free [4], benchmarking of the structure-function relationship in correlation networks remains ongoing. In many cases the generation of the co-expression network relies heavily on filtering and correlation measures of a snapshot of the cellular gene expression at a certain time or environment; further, correlation measures are almost always accompanied by the caveat that correlation “doesn’t imply causation.” As such, it can be difficult to discern noise from signal. Further complicating the analysis, it is known that gene co-expression is robust to transcriptional control changes [16,17]. As such, the set of genes identified as being co-expressed with others at a given time or state may include subgroups of genes under multiple levels of transcriptional control; further, it has been found that co-expressed genes tend to be robust toward change [18] (and thus more readily identifiable) when they have stable regulatory programs [17]. Structurally, it is known that scale-free protein-protein interaction networks are robust toward mutations unless those attacks are targeted at a hub node [4,10]. Another characteristic of these scale-free networks is modularity, or the tendency of the network to form modules. Theoretically, as the density of a cluster increases, the more connections and as such, redundancies it has toward single node deletion. It stands to reason, or at least to speculation, then, that dense clusters in a biological network may represent gene or protein cohorts that are functionally important due to this robustness, as the cell is programmed to be able to quickly and efficiently compensate for loss of expression. Thus, this research investigates the link between transcriptional robustness and cluster redundancy by focusing on dense clusters that can be readily identified using a varied density filter, rather than communities or motifs.

While adding in auxiliary data (such as incorporating Gene Ontology (GO) associations into clustering scores) may aid in finding true structures with biological impact faster, the problem remains that publicly available databases remain incomplete due to the vast array of possible functionality at the cellular level [19]. Many ontological databases contain a large amount of false positive information, remain incomplete, and/or may misrepresent data as a result of improper functional ontology descriptors. While these databases remain helpful resources, a method that is able to find structures with real biological implications in the network without incorporation of a prior bias lends itself toward a higher impact result. This search for a link between structure and function is currently the focus of many studies in network structure [9,20-24]; however, identification of these true biological processes or elements within a network currently has a finite upper limit that is often dependent on network size and complexity [25]. The issue remains that many networks built from high-throughput data are too large for current structure finding algorithms to find complex graph theoretic structures (such as graph partitioning, multi-way cuts, graph coloring, etc.) in reasonable time even with parallel computing resources at one’s disposal. The crux of this work therefore focuses on the identification of critical structures in a notoriously noise-heavy two-state network that can be implemented without access to large computational resources.

### Correlation networks

The application of network theoretical concepts to describe models of cellular systems in expression data remains in relative infancy and thus benchmarks are still being established [9-11]. In this study, correlation networks are used to capture relationships between probes. The correlation network is a graph model built of edges and nodes, where nodes represent gene probes and a set of sample expression levels for that gene, and an edge represents the level of correlation the two expression vectors. Different measurements of correlation have been used to build these networks, such as the partial correlation coefficient, well-suited for finding co-expressed motifs [12,26], the Spearman correlation coefficient, which best identifies non-linear relationships [27], or more commonly the Pearson correlation coefficient, which identifies linear relationships [11,28,29]. The network built from a dataset where all nodes (genes) are connected to each other is called a complete network, K_*n*_ (where *n* = the number of nodes/genes in the network). In K_*n*_ network, the number of edges is equal to n*(*n*-1)/2; this implies that in the case of datasets with a large number of genes, analysis of the K_*n*_ network can be computationally taxing when high performance computing options are not available. For example, a network made from 40,000 nodes will have almost 800 million edges. Thus, some type of thresholding or network filtering [25] is a common method used for network reduction.

The most straightforward method of thresholding involves removing edges with a low correlation (~0.00 in a Pearson correlation generated model). In larger networks, this threshold must become more stringent to maintain a size of network that can be quickly and properly analyzed. A threshold range of maximum ±0.70 to ±1.00 is typically used because it retains a coefficient of determination (variance) of at least 0.49. This indicates that correlations remaining within the network will represent genes whose expression levels can be described as approximately 49% dependent on each other’s expression. Carter *et al.* 2004 used this method of “hard” thresholding by correlation level and additionally used a p-value < 0.0001 threshold to ensure that only significant correlations had been retained [9]. Other methods that incorporate soft thresholding allow for variance in the actual correlation value based on distribution of node degree [29]; this method goes on to use topological overlap matrices to identify modules of functional significance with great accuracy. Pawitan *et al.* 2005 notes the need for multiple testing by FDR in their analysis of microarray statistics, noting that using a p-value solely in data cleaning can result in low sensitivity. Attempts to correct for this and other types of statistical concerns have been addressed in a variety of ways [30]. As previously stated, Carter *et al.* 2004 used a version of a network permutation test to reduce size [9].Other methods focus less on statistical significance of the correlations used, but look instead at other network characteristics. For example, in a 2004 comparison of multiple species data, Bergmann *et al.* kept the amount of genes to be analyzed relatively similar, resulting in a relatively constant size of network, i.e., it would be biased to compare networks of vastly different size [31]. Zhang *et al.* 2005 suggested that it may be most helpful to filter networks such that they fit a scale-free topology criterion, such that the linear regression of the log/log representation of the node degree distribution falls within an R [2] > 0.80 where R [2] measures the coefficient of determination [11]. Still other methods for network reduction include merging of common or commonly attributed nodes, helpful in a top-down approach. We acknowledge that different methods of thresholding may be appropriate for differing objectives and as such, the method to use should be decided upon on a per case basis until benchmarking studies can further suggest an appropriate optimized correlation model. It appears that all methods of network generation via some measure of correlation is able to return some measure of high impact result; suggesting the power of the correlation network and additionally a possible future need for benchmarking studies to investigate which measure is the most appropriate for which domain.

Typically, correlation networks are ideal for use in the analysis of relationships. Traditional methods for microarray analyses tend to miss by focusing on identifying lists of target genes based on differential gene expression, determined through a number of statistical tests over a two or more time-series snapshots. The advantage of the correlation network is the ability to capture relationships between gene pairs, and additionally between gene replicates, over time. The inherent ability of state comparison using differential co-expression has been used recently to identify complexes with discrete biological function in Alzheimer’s disease using network modeling [1]. Thus, the ability to represent relationships gives the correlation network a distinct advantage over traditional methods. However, correlation networks are notorious for having noise or unnecessary edges [32]; additionally, the volume of data to be analyzed remains a problem for users without access to parallel computing resources. As such, until the technology surrounding computational resources improves, other methods must be found to exploit the power of the correlation network by reducing the size and complexity of the problem (for instance, by network filtering, which looks to graph theoretic properties to reduce edge and node count [25]). In our research, we allow some noise to remain (what is left after thresholding and hypothesis testing) and show that network structure can identify causative genes by verifying that our results are indeed potential targets for further experimentation. This work is largely agreed upon as data-driven research [33], and as such, a typical hypothesis that describes specific goals of the work isn’t given. Informally, we are proposing a study in modeling gene expression via correlation network that identifies overlapping genes or gene products between modular structures in different states will reveal potential targets for further study in the aging mouse hippocampus. The results show that target identification via this method is able to uncover a small set of genes with major impact in the developing hippocampus from a large, highly dimensional set of high-throughput, *publicly available* data. Should this technique be applicable to the study of other diseases, it could possibly provide a low-cost, low-labor requirement method for identifying potential target genes in diseases with poorly understood mechanisms.

## Results

**Figure 1:**
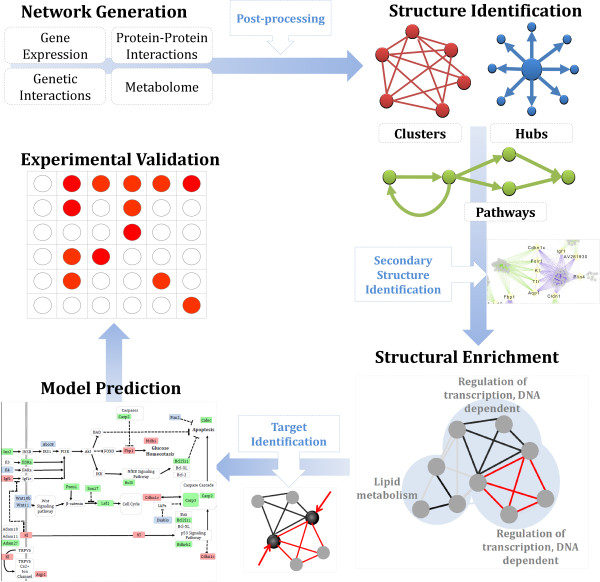
**A flowchart of the process described to build networks and find gateway nodes.** The first step is to create a K_n_ network base (where *n* = number of probes) on which layers of information are applied, including state data from gene expression correlation. The K_n_ network skeleton is then filtered and annotated to only include edges with correlations within threshold range, and each edge is annotated with its state. This results in graph *G* with two types of edges, an integrated network. Identification of biologically relevant clusters and gateway nodes follows, and functional annotation is then performed using Gene Ontology node enrichment and edge annotation. Once these target genes are identified, importance is determined via manual literature curation toward the experimental objective at hand, in this case, the implications of gateway nodes in the aging mouse hippocampus.

Data for network creation was collected and prepared according as described in “Network Creation” in the Methods section, and an overall description of the method is shown in Figure 1. After the young mouse networks (YNG) and middle-aged mouse networks (MID) networks were created and clustered, three integrated networks were generated: the union of clusters of density ≥65% from YNG and MID, the union of clusters of density ≥6 = 75% from YNG and MID, and the union of clusters of density ≥85% from YNG and MID. Gateway nodes were then identified from each of these three integrated networks (see “Structure identification” in the Methods section).

### Gateway nodes are not necessarily essential

Gateways were detected for each of the three networks and lethality of the gateway datasets was assessed. Table 1 shows the resulting gateway nodes from each network, as the Affymetrix ID, Genbank ID, and Gene Symbol. The cluster densities are shown in the 4-6^th^ columns; if a node is a gateway in the consecutive networks, the box contains the gatewayness score and is colored gray. There was not a significant loss of gateway nodes when switching from 65% to 75% filter (30 gateways to 26 gateways, respectively), but changing the cluster threshold from 75% to 85% resulted in a major loss (26 gateways to 4). Additionally, lethality fell for each increase in cluster threshold; 40% of the gateways were lethal in the 65 network, compared to 38% in the 75 network and 25% in the 85 network. Significance testing was performed as described under Methods – Simulated Networks; compared to simulated Erdos-Reyni and Scale-free networks of similar size, these gateway nodes were found to be significant for P-value <0.0005 at 65% and 75% and P-value <0.05 at 85%. This indicates that regardless of threshold, gateway nodes do not tend to represent essential genes when compared to other node ranking measures. In their 2001 study, Jeong *et al.* found that yeast hub nodes tend to have a 60% lethality rate [8], and studies in correlation network centrality has shown that degree, betweenness, and closeness can be an indicator of essential gene likelihood (~40%), but with less clarity than what has been found in protein-protein interaction networks [7].

**Table 1:** The gatewayness of nodes at 65%, 75%, and 85% cluster density

**Affymetrix ID**	**GenBank accession #**	**Gene symbol**	**65% cluster density**	**75% cluster density**	**85% cluster density**	**MGI phenotype “lethality”**
160799_at	AW060549		100.00%	100.00%		
162085_r_at	AV334165	Actr10	100.00%	100.00%		
95552_at	U49861	Dio1	100.00%	100.00%		Yes
96918_at	AI790931	Fbp1	100.00%	100.00%		Yes
97546_at	AF072127	Cldn1	100.00%	100.00%		Yes
102089_at	Y10521	Matn3	100.00%	100.00%		
99876_at	U29056	Sla	100.00%	100.00%		
96483_at	C80828	Dhrs7b	100.00%	100.00%		
102763_at	AF064748	Plin4	100.00%	100.00%		
160733_at	AI035317	Akr1c21	100.00%	100.00%		
97523_i_at	×02578	Amy2a4|2a5|2b	100.00%	100.00%		
162391_r_at	AV260455	Ltc4s	100.00%	100.00%		
92289_at	×58289	Ptprb	100.00%			Yes
161714_f_at	AV250133	Maoa	100.00%	100.00%	100.00%	
160504_at	AI197077	Ceacam12	100.00%			
93809_at	U41736	Aup1	100.00%	100.00%		
97238_at	AW209238	Tacc3	100.00%	100.00%		Yes
160772_i_at	AW214428	Slu7	100.00%	100.00%	100.00%	
96004_at	AI851641	Sri	100.00%	100.00%		
92283_s_at		Il4	100.00%			Yes
98803_at	L77247	Zfp354a	100.00%			
97508_at	M29462	Mdh1	100.00%	100.00%		Yes
95546_g_at	×04480	Igf1	51.97%	51.59%		Yes
162101_f_at	AV290649	Mylpf	51.67%	50.86%	32.20%	Yes
161622_f_at	AV356315	Lman1	50.00%	49.14%	49.15%	
161229_at	AV261930		48.82%	48.41%		
162302_f_at	AV035020	Folr1	23.20%	21.16%		Yes
93330_at	L02914	Aqp1	23.20%	20.91%		Yes
100956_at	AB005141	Kl	21.91%	20.40%		
95350_at	D00073	Ttr	20.62%	18.89%		
95471_at	U22399	Cdkn1c	20.10%	18.64%		Yes
		P-Value	5.53E-15	3.63E-12	0.028137	
		Significance	***	***	*	^1^

^1^Significance: *p-val <= 0.05, **pval<=0.005, ***pval<=0.0005.

If we examine this table further, a pattern seems to emerge between those nodes whose gatewayness is shared (0-99%) or solely the responsibility of that node (100%). If we compare lethality between shared vs. sole gateways, we find the following lethality in Table 2.

**Table 2:** Sole and shared gateway node lethality for each cluster density threshold

**Gateway type**	**65% density**	**75% density**	**85% density**
**Sole**	31.8%	27.8%	0%
**Shared**	55.6%	55.6%	50%

Future studies include further examining the properties of these gateway nodes to determine if a subset within them has relevant biological properties. This includes identifying whether or not shared gateway nodes, which appear to have a higher lethality rate than typical central nodes in a correlation network, exhibit this property in other temporal datasets.

**Figure 2:**
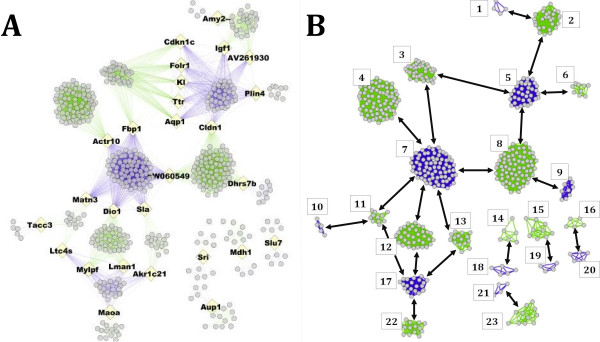
**The integrated graph *G *with two edge types/states (2A) shows individual clusters from differing networks that are indeed connected by one or a few nodes between clusters.** The gateway nodes, highlighted in 2**A** as larger yellow diamond nodes, are not hubs in the traditional sense (i.e., as in an interactome). Figure 2**B** represents the network with the nodes removed (arrows representing the clusters they connect) highlights that this special sets of nodes becomes a minimum cut set between the YNG and MID networks.

Visual inspection of the integrated graph *G* with two edge types/states (Figure 2a) confirms that individual clusters from differing networks are indeed connected by one or a few nodes between clusters. The gateway nodes, highlighted in Figure 2a as larger yellow diamond nodes, are not hubs in the traditional sense (i.e., as in an interactome). Hubs within correlation networks are typically found to exist within large dense clusters of genes, and as such some gateway nodes are also hub nodes in their original networks [34]. In the biological sense, these are genes or probes that exhibit correlated expression to one set of genes in one state, and are correlated to an almost completely different set of genes in another state (unless they are connected to other gateways in their set). When these nodes are removed from the network, they completely disconnect the clusters; when the domain is defined as two subsets or clusters. Figure 2b represents the network with the nodes removed (arrows representing the clusters they connect) highlights that this special sets of nodes becomes a minimum cut set between the YNG and MID networks.

#### Gene ontology enrichment reveals different functions in young and middle aged mouse hippocampus

Gene Ontology enrichment was performed as described in Methods on each cluster in the integrated network without gateway nodes. Clusters are numbered as shown in Figure 2b. No significant enrichment was found for clusters 1,9,10, 13–16, and 18–23. Enrichment scores for the other clusters can be found in Additional file 1: Table S1. Individual cluster enrichment revealed mostly metabolic/biological process results, but nothing that indicates a role in hippocampal aging. Cluster 3 (a YNG cluster) was enriched in terms apoptosis, programmed cell death, death, and regulation of cell death. A 2006 study in hippocampal aging have found that neuronal degeneration occurs in senescence accelerated mice, but further investigation of apoptosis relating to morphological changes in neurons was not able to tie pro- or anti-apoptotic factors to this phenomenon [35]. A later study in *Klotho* deficient mice, typically used as models for CNS aging and exhibiting signs of cognition impairment, found that apoptotic and anti-apoptotic protein expression in the brain is changed compared to WT, specifically that pro-apoptotic factor *Bax* and apoptotic inhibitor *Bcl-XL* are reduced in Kl-/- mice [36]. Interestingly, *Klotho* is a gateway gene identified in our 65% and 75% density networks.

Cluster 4 (YNG) was found to be enriched in many terms, but the relevant among them was found to be tissue morphogenesis (over-enriched), and embryonic development genes (under-enriched), and genes relating to development of anatomical structures. The two main MID clusters with non-biological process GO enriched terms were clusters 5 and 7. Cluster 5 was over-enriched with 57% of terms that included the term “regulation”, and cluster 7 displayed under-enrichment in cell-cycle genes, phosphorylation, and regulation of cell proliferation and developmental processes.

Individual cluster results returned some interesting terms, but it was noted some of the clusters from the same networks exhibited similar terms but that were not significantly enriched. As such, we performed GO Enrichment as well on the entire YNG and entire MID networks independently. Gateway nodes were included in both the YNG and MID network enrichments as well. The results of this enrichment are found in Table 3. We find again that the YNG network is over-enriched in apoptosis, programmed cell death, and maintenance of homeostasis. The MID network was found to be enriched in terms that suggested a change in metabolic activity (under-enrichment of positive regulation of metabolic process) and changes in transcription. Based on this information, we can conjecture that functions involved in the young hippocampal process involve programmed cell death, and the middle-aged hippocampus involves a stronger grasp on transcriptional control.

**Table 3:** GO Enrichment of YNG and MID clusters in the integrated network

**Age**	**Annotation**	**GO Term ID**	**Observed**	**P-value**	**Enrichment**
YNG	positive regulation of biological process	GO:0048518	43	0.0300	up
	response to stimulus	GO:0050896	41	0.0492	up
	plasma membrane part	GO:0044459	25	0.0329	up
	small molecule metabolic process	GO:0044281	20	0.0471	up
	apoptosis	GO:0006915	18	0.0356	up
	programmed cell death	GO:0012501	18	0.0356	up
	homeostatic process	GO:0042592	11	0.0226	up
	positive regulation of cell death	GO:0010942	10	0.0343	up
	positive regulation of apoptosis	GO:0043065	9	0.0150	up
	positive regulation of programmed cell death	GO:0043068	9	0.0150	up
MID	organelle part	GO:0044422	23	0.0482	up
	intracellular organelle part	GO:0044446	21	0.0338	up
	catalytic activity	GO:0003824	16	0.0222	up
	plasma membrane	GO:0005886	16	0.0476	up
	regulation of biological quality	GO:0065008	15	0.0033	up
	plasma membrane part	GO:0044459	14	0.0100	up
	positive regulation of metabolic process	GO:0009893	11	0.0221	down
	protein complex	GO:0043234	11	0.0280	up
	regulation of transcription from RNA polymerase II promoter	GO:0006357	10	0.0386	down
	transcription from RNA polymerase II promoter	GO:0006366	10	0.0386	down

Observed column represents the number of genes in the network with the specified annotation.

Gene Ontology edge annotation allows for visualization of functional relationships between gateways.

**Figure 3:**
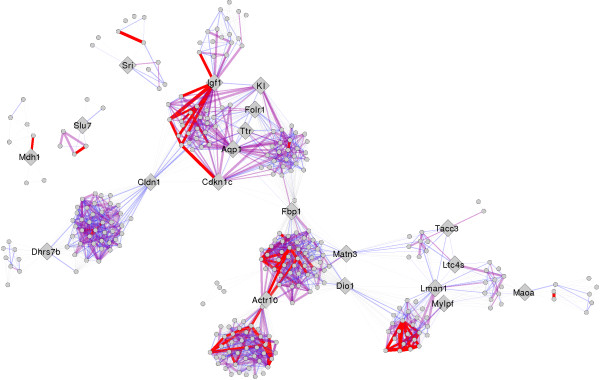
**The GO edge annotated network.** The GO edge annotated network where only edges with a score of 0 or higher (maximum score = 11) are opaque, and the edge with represents this score (the thicker the edge the higher the score. Edge color represents score in the following ranges: gray ➔ -12 to 0, blue ➔ 1 to 3, purple ➔ 4 to 7, red ➔ 8 to 11. Gateway nodes are represented as large gray diamonds, and other nodes are reduced in size for easier viewing. Immediately visible are thick red edges connected to gateway nodes *Igf1, Cdkn1c,* and *Actr10*.

In addition to traditional ontology enrichment performed on the nodes, we also performed edge ontology annotation using GO’s Biological Process tree. The method identifies common parents in the GO parent–child structure such that each edge is annotated with the common parent and a score for that relationship based on how far apart or close the nodes are in relationship to the parent, and the depth of the parent in the tree [20]. This method focuses on annotating the edges of the network and thus removes some noise by only looking at pairwise relationships between genes, with a score of 0 or lower reflecting less important relationships and scores > 0 representing increasing importance of that relationship in the GO. The resulting integrated network after the edge annotation method was applied is a network in which edges are included 1) only if they were in the original network and 2) only if there was a common parent found between the two nodes in the network. The GO edge annotated network is shown in Figure 3. In this network, only edges with a score of 0 or higher (maximum score = 11) are opaque, and the edge with represents this score (the thicker the edge the higher the score. Edge color represents score in the following ranges: gray ➔ -12 to 0, blue ➔ 1 to 3, purple ➔ 4 to 7, red ➔ 8 to 11. Gateway nodes are represented as large gray diamonds, and other nodes are reduced in size for easier viewing. Immediately visible are thick red edges connected to gateway nodes *Igf1, Cdkn1c,* and *Actr10*. Edges with the next lowest visible (purple) edges of importance appear to be *Klotho* and *Aqp1.* To determine the most likely functional candidates according to functional association, we average the total depth score of all edges connected to a given gateway; the results are shown in Table 4. Nodes included or connected to cluster 3, 4, 5 or 7 are in italics.

**Table 4:** Ranked gateways

**Gateway name**	**GO annotated network degree**	**Average GO depth**
*Igf1*	*42*	*3.190*
*Aqp1*	*60*	*2.017*
Mdh1	5	1.800
Tacc3	9	1.444
Sri	7	0.714
*Cdkn1c*	*51*	*0.309*
*Lman1*	*47*	*0.170*
*Kl*	*56*	*-0.393*
Ltc4s	22	-0.682
Maoa	13	-0.769
*Folr1*	*68*	*-1.044*
*Cldn1*	*67*	*-1.254*
Slu7	6	-1.500
Mylpf	39	-1.769
*Ttr*	*53*	*-2.036*
*Dio1*	*77*	*-2.338*
*Fbp1*	*76*	*-2.368*
Dhrs7b	35	-2.657
*Actr10*	*79*	*-3.051*
*Matn3*	*55*	*-3.345*

The gateways that turn up when the GO Edge annotation method is applied, including their degree in the annotated network, and their average GO depth (only for immediately adjacent edges).

Based on this knowledge from our GO Enrichment studies and the edge annotation analysis, we have a few processes on which to focus (apoptosis and transcriptional regulation) and some genes that are possibly playing a role. The genes that are involved in the most likely clusters of relevance (3, 4, 5, and 7) are scattered among the GO edge annotation list, so we can rank those in terms of most likely importance. Potential candidates for targeting changes in mouse hippocampal aging, then, become the following in decreasing order: *Igf1, Aqp1, Ckdn1c, Lman1, Kl, Folr1, Cldn1, Ttr, Dio1, Fbp1,* and *Actr10.*

## Discussion

**Figure 4:**
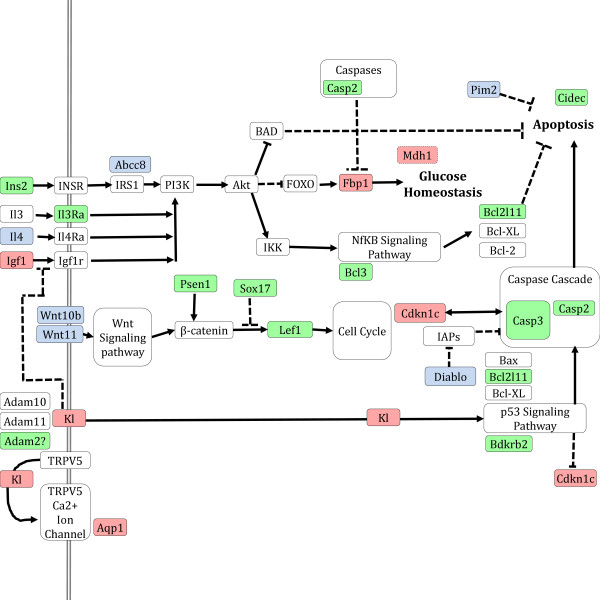
**The model that was created using interactions between gateway nodes and cluster.** The grey double edged line is the cell membrane, red nodes are gateway nodes, green nodes are young nodes, blue nodes are aged nodes, and white nodes are added in to connect these nodes. Edges with a flat top indicate inhibition and edges with arrows indicate directed interaction.

Further investigation of these candidate gateway nodes in literature reveals that there is definite potential for systematic impact of these gateway nodes in the aging mouse hippocampus. We include a model (Figure 4) that includes any relevant pathway/interaction information of these genes, manually curated from literature and intended to highlight any possible links between the gateway nodes proposed. Genes/gene products highlighted in red are gateway nodes, and genes/gene products highlighted in blue and green represent genes that are also in the integrated network model, in the middle-aged or young clusters, respectively.

*Igf1* is a homolog of *Ins*, both of which have been implicated in multiple adult hippocampal development as crucial for normal aging and health in mice [35,37,38] and rats [39]. *Igf1,* when bound to its receptor *Igf1r*, activates the PI3K-Akt Pathway, which has been found to be critical for neuronal axon growth [40]. Additionally, the Pi3k-Akt pathway is directly upstream of apoptosis and glucose homeostasis (as shown in Figure 4). *Igf1* is a gateway node between clusters 2 (YNG) and 5 (MID). *Igf1* in particular has been found as a critical component of aging in mouse models – *Igf1* deficient mice were found to have reduced brain sizes suggesting an *Igf1* role in axon maturation [41]. Ames dwarf mice with *Igf1* deficiencies exhibit longer lifespan and studies have speculated that *Igf1* and growth hormone (*GH*) are responsible for structural integrity in the brain [42]. A 2008 review of *Igf1* related literature found that it is actually pathways involved in neurotrophin signalling downstream of the *Igf1* receptor that plays a role in brain aging and suggests it as an aging related target [43].

The evidence supporting the role of *Aqp1,* or aquaporin 1, in hippocampal development is less clear, however; it has been shown in *Aqp-/-* mutants that neuron excitability is diminished [44]; it has also been shown that *Aqp1* expression is higher than normal in patients with Alzheimer’s disease (AD) [45]. It is unclear the role *Aqp1* may have in pathways associated with apoptosis and regulation in the hippocampus. *Aqp1* is a gateway node connecting clusters 3 (YNG) and 5 (MID).

*Cdkn1c,* a cyclin responsible for inhibiting proliferation, is usually associated with cell cycle regulation, but also finds roles in programmed cell death. It connects clusters 3 and 5 with *Aqp1*. Particularly, *Cdkn1c* has been implicated as a cyclin-dependent kinase that is active during embryogenesis, and *Cdkn1c-/-* mice have major developmental problems involving differentiation [46]. A study in HeLa cells revealed a link between *Cdkn1c* and increased expression caspase-3 encoded by *Casp3,* thereby implicating it as a pro-apoptotic [47] (shown in Figure 4).

Knockouts of the gene *Kl* result in mutants exhibiting growth deficiencies, shortened lifespan, and a myriad of other issues including bone deficiencies and hardening of the arteries [36]. Conversely, Klotho over-expression mutants live on average 20-30% longer than wild-type [48]. Further, *Kl* has been found to have a role as an inhibitor in the Insulin and IGF signalling pathway [49] (modified role shown in Figure 4). Klotho connects cluster 3 and 5 with *Cdkn1c* and *Aqp1*. Acting as a membrane and a secreted protein, Klotho can interfere with upstream receptors in the *Ins/Igf* pathway, resulting in lowered activity with *PI3K*[49]. Many more studies have implicated *Kl* as an ‘aging’ gene [36,48-53]; it is highly expressed in first the kidney and then the brain in mouse models, and also results in abnormalities such as hypogonadism, ectopic calcification, epidermal atrophy, emphysema, hearing loss, elevated Vitamin D and calcium levels, and neurodegeneration [51]. Neurodegeneration in *Kl-/-* mice has been found as increased rate of programmed cell death [51] and mutants show cognitive impairment in recognition and fear testing [52].

Like *Kl, Cdkn1c,* and *Aqp1, Folr1* connects clusters 3 (YNG) and 5 (MID). While the average of its GO edge annotations is lower than all the previous gateways discussed at -1.044, it may still have functional relevance due to its high number of annotated connections in the network (degree = 68, the top ranking node in terms of degree in the GO edge annotated network). As a gateway, it is the final of the 5 gateways between clusters 3 (YNG) and 5 (MID). However, studies of *Folr1’s* role in the murine hippocampus remains limited. A 2010 study in 12-month-old mice transfected with human Tau23 protein 51 genes total were found to be up- or down-regulated by the phosphoprotein [54]; *Folr1* was found to be the second highest up-regulated gene compared to controls with the a fold change score of 7.18 [54]. Other genes in the 51 gene dataset included gateway genes *Aqp1* (up, FC = 6.17), *Kl* (up, FC = 3.43), *Cldn1* (up, FC = 2.89), *Cdkn1c* (up, FC = 2.27), and *Igf1* paralog *Igf2* (up, FC = 2.03) [54]. In total, 6 of the 11 predicted target gateway genes are identified as regulated by human *hTau* encoded Tau23 protein. Another study of human Tau knockouts in mice found that mutants exhibit suppressed cell growth and neuronal counts increased compared to wild type [55], and suggests that Tau can cause activation of programmed cell death in neurons of the hippocampus by cleavage of *Casp3*[56]. This suggests the possible role of a murine *hTau* homolog in regulation of the control of normal murine hippocampal development (shown in Figure 4).

The last gateway connecting clusters 3 (YNG) and 5 (MID) is *Ttr,* is also known as Transthyretin. Few studies have been performed on *Ttr* in the normal developing hippocampus, but *Ttr* has been studied in the context of Alzheimer’s Disease (AD) as an interactor with amyloid-β protein [57]. *Ttr* also interacts with hormone thyroxine (T4). In one of the AD –related studies, it has been found that neuronal degeneration was accompanied by increased levels of transthyretin [57]. This is verified by a 2011 study that found *Ttr* variants to be the highest up-regulated gene (FC = 57.04, 39.52, 32.01, and 23.4 ) in mice with HuD-Tg overexpression, which is involved in neural growth and connectivity [58].

*Dio1*, a gateway node between clusters 12 (YNG) and 7 (MID) is included in the potential target list for its membership in GO Enriched cluster 7. Deiodinase-1 as well has not been studied extensively for its role in the developing hippocampus, although it should be noted that it also interacts with thyroid proteins T3 and T4, similar to Transthyretin. A 2012 study in *Danio rerio* revealed that alterations in T3 and T4 levels in larvae resulted in increased expression of *Dio1* and paralog *Dio2,* while levels of *Ttr* was down-regulated. While these studies are not directly conducive to how these genes affect hippocampal development, they offer a possible link between gateways and the thyroid system [59].

*Fbp1,* gateway node for clusters 3 (YNG) and 7 (MID), is typically associated with glucose generation, but a 2005 study linked *Fbp1* in *Saccharomyces cerevisiae* to aging and oxidative stress [60].

*Actr10,* the sole gateway node for clusters 4 (YNG) and 7 (MID), plays a role in actin and microtubule movement. *Cldn1,* the sole gateway node for clusters 5 (MID) and 8 (YNG), is involved in tight junction formation. Neither of these gateway nodes has been studied in the context of the aging mouse hippocampus.

Readily detected network structures such as hubs, clusters, pathways, or bottlenecks, are measured typically in a static network. While these can be measured in an integrated network with relative ease as well, the relationship between structure and function in the integrated network does not necessarily hold in the integrated network; at the very least, this relationship has not been explored. The gateway node offers a way to measure relevant structure created by the integrated network model, and a major purpose of this study is to investigate potential biological relevance of this structure. The results suggest that gateway nodes may represent some sort of developmental pivot in aging mouse studies.

## Conclusion

Studying relationships between genes and gene products provides an important perspective in the study of biological function. Network models provide an excellent tool for modelling intergenic relationships associated with a particular domain. In this research we have proposed a formal method for the identification of critical elements associated with a biological process such as aging. This method explores the temporal similarities and dissimilarities among relationships at different stages of aging in the mouse hippocampus. Elements play significant roles in the transitional process among those stages are characterized using graph theoretic properties. We show that these elements we call gateway nodes represent genes that link critical functions at different stages of development. A majority of these nodes have previously been identified as elements associated with normal aging, which serve as a validation to our proposed approach. The remaining elements captured by gateway analysis correspond to genes previously linked to aging or aging-related processes outside of the hippocampus. The strength of the proposed method lies in its ability to model biological systems at various states and exploring changes associated with certain diseases or the degradation of cellular health.

## Methods

The proposed overall method is described in visual detail in Figure 1. The first step is to create a K_n_ network base (where *n* = number of probes) on which we apply layers of information, including state data from gene expression correlation. (Redundant genes in the dataset were allowed to remain.) The K_n_ network skeleton is then filtered and annotated to only include edges with correlations within threshold range, and each edge is annotated with its state. This results in graph *G* with two types of edges, an integrated network (shown in Figure 1). This approach method can be expanded to include multiple types or conditions and is planned for future work. Identification of biologically relevant clusters and gateway nodes (described below) follows, and functional annotation is then performed using Gene Ontology node enrichment [61] and edge annotation [20]. Once these target genes are identified, we manually examine their importance toward the experimental objective at hand, in this case, the implications of gateway nodes in the aging mouse hippocampus.

### Network creation

Data Series GSE5078 generated by Verbitsky *et al.* in 2004 [62] was obtained from NCBI’s Gene Expression Omnibus (GEO) website (http://www.ncbi.nlm.nih.gov/geo/) in December 2009. The C57BL/6 mice used in this dataset were separated into two age groups – young and middle-aged – and were untreated and expression data was drawn from hippocampus after latency testing in the Morris water maze [62]. The dataset was separated into 2 month old samples (YNG) versus 15 month old samples (MID), for 2 states total. Probes with undetectable expression or missing values were not used in the analysis; probes with any value of expression were allowed to remain including those with weak values. As indicated in Verbitsky *et al*. 2004 and their supplemental material, data were normalized using RMA techniques. The values given in the GEO Series Matrix Files were used exactly as presented in the table itself. This series was chosen because of relation to aging in the mouse brain, mouse model type, state number and sample size.

Networks were created in parallel by pairwise computation of Pearson Correlation [63] (*ρ*) for each possible combination of probes within the dataset on the University of Nebraska at Omaha’s Blackforest computing cluster. Nodes in the network represent probes and edges represent the weighted correlation of each gene and an associated p-value. Correlations with p-value < 0.005 (Student’s T-test [63]) were not considered statistically significant and thus those edges were thrown out. Networks were then filtered to a correlation threshold of 0.85 ≤ *ρ* ≤ 1.00 to capture only very highly correlated expression values; this threshold was chosen to capture only genetic relationships where 70% or more of one genes behavior could be ascribed to the behavior of another gene (R [2] of the proposed lower correlation bound of 0.85 is 72.25%). After duplicate edges and self-loops were removed, both networks created were found to adhere to a power-law degree distribution and exhibit qualities of a modular network (networks are included in .sif format in Additional file 2).

### Structure identification

#### Clustering

For this particular proof-of-concept, cluster identification was performed Cytoscape plug-in AllegroMCODE v2.0 [64] on each network with settings set at Degree Cutoff = 4 and K-Core = 4 to eliminate K_3_ cluster identification. Clusters with a density of <65% were thrown out. AllegroMCODE was chosen for its ability to identify dense clusters within a large network quickly; this structural characteristic has been found to be representative of probable biological function in correlation network studies [11,29]. As gateway node identification is largely dependent on node inclusions within a cluster, we used clusters at 65% + density, 75% + density, and 85% + density to identify gateway nodes. Often increasing the threshold in this way removed only a few clusters from 65% density threshold to 75%, but these minor cluster removals also had a big impact on the type and number of gateway nodes present. Further examining the effects of correlation and cluster thresholding on gateway nodes is planned for future studies, as well as additional clustering methods. Recently, clustering methods have been assessed on gold standard complexes in the known *Saccharomyces cerevisiae* interactome and it has been discovered that different clustering methods have different performance in terms of cluster accuracy and sensitivity [14,15]. These studies reveal that MCODE [13] (AllegroMCODE’s base methodology) has a tendency to over-predict clusters in terms of size but methodologically is able to find dense clusters with great accuracy; while this may be inefficient for detecting protein complexes, this is ideal for identifying clusters in correlation networks, that do not necessarily have to correspond to co-functional relationships, just co-expression. Further, while this work acknowledges the capability of other clustering methods, it remains that an assessment of all possible methods is out of the scope of this particular study.

### Gateway nodes

In earlier studies, it has been empirically observed that when two murine networks of same tissue but different temporal states are compared, there is little overlap of dense network clusters [7,20,25]. However, there are several nodes, or “gateway nodes”, that connect the clusters from different states individually or as a group. Preliminary work with these gateways suggests they may point to important genes for the observed transition between temporal states. Here, we define a formal method for identifying these nodes between two states in a temporal correlation network, and formally define a “gateway node” as a gene identified by this approach.

The concept of gateway nodes is a relaxed notion, or a mathematical generalization, of the well-established concepts of cut-nodes and node cut sets in graph theory. Given a general connected graph, a node is defined as a cut node if its removal leaves the graph disconnected. Similarly, a set of nodes in any connected graph define a node cut set if the removal of the nodes in the set leaves the graph disconnected. A cut node is a special case of a node cut set where the set contains only one node. In the context of integrated correlation networks, we are interested in identifying a small set of nodes that play in a significant role in connecting two sets of highly-dense sub-graphs of a graph that represents the underlying relationships obtained from multiple correlation networks.

Consider two correlation networks, represented by graphs *G1* and *G2*, which reflect correlation relationships among genes of same tissue and organism at various states. Let undirected graph *G1* = *(V, E1)* represent state 1 and let undirected graph *G2* = *(V, E2)* represents state 2, such that graphs *G1* and *G2* share same node set *V = {v*_*1*_*, v*_*2,*_*…, v*_*n*_*}* with different edge sets *E1* and *E2*. For each graph, we identify clusters (highly dense sub-graphs); for example, Cluster *X* represent some dense sub-graph in *G1* where *V(X)* ⊆ *V(G1)* and *E(X)* ⊆ *E(G1)*, and Cluster *Y* represent some dense sub-graph in *G2* if *V(Y)* ⊆ *V(G2)* and *E(Y)* ⊆ *E(G2).* To obtain the gateway nodes associated with clusters *X* and *Y*, we first form an integrated graph *G’* such that *G’* = (*V,(E1* ∪ *E2))*, and for a given node *s* in *V,* we identify *E*_*s*_*(X,Y)* as the set of edges connecting *s* to any node in the set *V(X)* in graph *G1* or any node in the set *V(Y)* in graph *G2*. The goal is to identify a set of gateways that connect the nodes of Cluster *X* and the nodes of Cluster *Y*, both now present in the integrated graph *G’*. Hence, for the two clusters *X* and *Y* in *G’*, we identify the subset of nodes *S* between clusters *X* and *Y* such that the set *S* = *V(X) ∩ V(Y)*. Since *S* is the intersection of the two sets of nodes *V(X)* and *V(Y)*, in the subgraph induced by *V(X)*∪*V(Y)* in *G’*, every connection path from a node in *X* to a node in *Y* has to go through one of the nodes in *S*. In other words, the nodes of *S* represent all the gateways connecting the two clusters *X* and *Y* in *G’*. Also, since the goal is to identify a relatively small set of nodes responsible for most of the connections between the two clusters, we insist that the size of *S* is always less than half the size of either *V(X)* or *V(Y)* in order to identify any node in *S* as a gateway (or, $\left|S\right|\leq \min\left\{\frac{\left|X\right|}{2},\frac{\left|Y\right|}{2}\right\}$). This restriction will also guarantee that the two clusters *X* and *Y* and sufficiently different and eliminate the scenario of having the trivial case where *V(X) = V(Y) = S*. To determine the gatewayness of each node *s* in *S,* that is its role in connecting nodes of *X* to nodes of *Y* in the integrated graph *G’*, or the amount of impact it has in the “transition” from one state to another, we define the following metric:

$$\mathrm{gatewaynes}s_{s}=\frac{\left|E_{s}\left(X,Y\right)\right|}{\left|\mathrm{Es}\left(X,Y\right)\right|}$$

Where *E*_*S*_*(X,Y)* is defined as the set of edges connecting any node in *V(X)-S* to any node in *V(Y)-S* in *G’*. Thus, we are essentially identifying the total number of edges connecting the nodes of *X* to the nodes of *Y* through the node *s*, and comparing that to the total number of edges connecting the nodes of *X* to the nodes of *Y* through every node in *S*. Note that edges connecting two nodes in the set *S* are excluded from calculation *E*_*S*_ as they are not edges that connect cluster nodes to a gateway node. If two clusters *X* and *Y* are connected by though a set *S* of size 1, the gatewayness for that only node *s* in *S* will be 1.00, or have 100% gatewayness.

#### Simulated networks

Simulation analyses were used to compare the significance of randomly generated networks to the networks used in these studies via two methods. Two types of networks were simulated using iGraph functions in R: Erdos-Reyni random networks and Scale-free networks. The simulated Erdos-Reyni networks were generated using the “erdos.reyni.game” command and networks were generated with 12,300 nodes using a 10/10000 edge probability parameter, yielding networks that are randomly distributed with regards to degree and of similar size to the YNG and MID networks. These networks by nature do not contain clusters; clustering via AllegroMCODE on these networks revealed indeed that no clusters were generated. Gateway node analysis was then performed considering each network individually and density requirements were not enforced. The result indicated that each node (12,300) in both networks was determined to be a gateway node with a uniform gatewayness null distribution; significance testing using the student’s t-test comparing actual gateways versus random network gateways found that all gateways in the YNG vs MID network were significant for P-value <0.05.

The simulated Scale-free networks were generated using the “static.power.law.game” command in iGraph and networks were generated to match the YNG and MID network sizes (respectively named “YNGSIM” and “MIDSIM”). Both networks were generated Exponent Out and In values of 2.5. Clustering via AllegroMCODE on these networks under previous standards (K-Core of 4 and Degree Cutoff of 4) revealed no clusters; Default parameter clustering revealed one large cluster per simulated network. Gateway node analysis was then performed considering each cluster individually and density requirements were not enforced. The result found 173 gateway nodes with a uniform gatewayness null distribution; significance testing using the student’s t-test comparing actual gateways versus random network gateways found that all gateways in the YNG vs MID network were significant for P-value <0.05. These values are reflected in Table 1.

### Functional analysis

#### Essential gene/lethality assessment and enrichment

The MGI Marker to Phenotype Annotations file (ftp://ftp.informatics.jax.org/pub/reports/MGI_PhenotypicAllele.rpt) from the Mouse Genome Informatics database [65] (MGI) was downloaded on January 02, 2013. For each node in the network, we determined in an *in vivo* knockout or knock-in mutation had been performed on that gene. If that mutation had been performed and caused any phenotype containing the word “lethality,” that gene was annotated as an essential gene. Using this we can perform basic lethality an enrichment analysis to determine the log-odds ratio enrichment of lethal genes in hub nodes versus the rest of the network. Enrichment is performed as stated below:

$$\mathrm{Enrichment}=\mathrm{lo}g_{2}\left(\frac{b/n}{B/N}\right)$$

Where *b* = count of lethal genes in test set, *n* = total count of genes in the test set, *B* = count of lethal genes in background set, and *N* = total count of genes in the background set. P-value was determined by performing hyper geometric distribution on the enrichment scores.

### Gene Ontology (Node) Enrichment

All Gene Ontology (node) Set Enrichment analyses were performed using GeneTrail [61] (http://genetrail.bioinf.uni-sb.de/) using Gene Symbols from the *Mus musculus* genome. Only manually curated annotations from the Gene Ontology were considered, with a p-value of <0.05 and no corrections applied. Background set used was the entire set of known mouse genes.

### Gene Ontology edge annotation

Gene Ontology edge annotation was performed using the methods as described by Dempsey *et al.* in [20]. Briefly, this method iterates through every edge in the integrated network and identifies the GO terms associated for each node pair connected by every edge. The method then identifies the deepest common parent (DCP) of those two nodes within the tree, how deep the DCP is from the tree root (depth) and how far many hops the nodes are apart from the DCP (breadth). The edge is then annotated with a GO term (the DCP) and a score (depth – breadth). Studies using this method have shown that this method supplements traditional GO enrichment by capturing missing GO annotations and revealing functional association based on edges, not nodes [20]. This is critical for network study, as two clusters may have the same amount of nodes, but a different amount of edges.

## Competing interests

The authors declare that they have no competing interests.

## Authors’ contributions

KD carried out network creation and analysis experiments. KD and HA conceived of the definition of the gatewayness node. KD and HA conceived of the study and participated in their design and coordination. Both authors read and approved the final manuscript.

## Supplementary Material

Additional file 1: Table S1 Gene Ontology enrichment scores for clusters, including term count, p-value, and up/down enrichment. Click here for file

Additional file 2 **The two threshold and p-value filtered correlation networks before clustering.** Gene IDs come from GEO Platform GPL81 (Affymetrix Murine Genome U74A Version 2 Array). These can be visualized using any network visualization software GUIS (i.e. Cytoscape) or manipulated in R using the *igraph* library. Click here for file
